# The Bradford and Other Competitions

**Published:** 1912-03-09

**Authors:** G. L. Desmond Hall


*To the Editor of* The Hospital.

### The Bradford and Other Competitions.

*To the Editor of* The Hospital.

Sir,—I have read with great interest your article urging that something should be done to reform the method of assessing hospital competitions, and, noticing that a discussion is invited, I venture to write to you on the subject.

I am rather surprised that you have made the East Sussex Hospital Competition your casus belli, because I am convinced—although an unsuccessful competitor—that the best design has been accepted. I am of opinion that an inquiry into the recent competition for the new Royal Infirmary, Bradford, would be far more to the point. The result of this competition was made public exactly twenty-two days after the drawings had been sent in. I believe about fifty-four architects competed, who could not have sent in less than nine or ten strainers each. Now, to look at the matter in a common-sense way, does it not seem impossible that the assessor could have gone into the various schemes carefully, made his award (which would have to be submitted in turn to a committee), and announced the result to the Press within so short a time? Moreover, I am told by a competitor who has seen the block plan of the winning design that the winner had disregarded the conditions and treated his plans as for a level site, whereas there was a fall of about fifty feet across the site.

It is preposterous that architects should be called upon to give their time and energy in preparing plans, when there is no guarantee that the conditions—in which it is specifically stated that any deviation from these particulars will disqualify the competitor—shall be strictly adhered to by the assessor. Such adjudications point to one of two things—either the assessor is incompetent, or something worse. In face of this I think an inquiry would be fully justified.—Believe me, yours truly,

March 5. G. L. Desmond Hall.

